# Applications of Information Theory to Epidemiology

**DOI:** 10.3390/e22121392

**Published:** 2020-12-09

**Authors:** Gareth Hughes

**Affiliations:** SRUC, Scotland’s Rural College, The King’s Buildings, Edinburgh EH9 3JG, UK; gareth.hughes@sruc.ac.uk

This Special Issue of *Entropy* represents the first wide-ranging overview of epidemiological applications since the 2012 publication of *Applications of Information Theory to Epidemiology* [1]. The Special Issue comprises an outstanding review article by William Benish [2], together with 10 research papers, five of which have been contributed by authors whose primary interests are in phytopathological epidemiology, and five by authors primarily interested in clinical epidemiology. Ideally, all readers will study Benish’s review—it is just as relevant for phytopathologists as it is for clinicians—and then clinicians and phytopathologists will take advantage of the opportunity to read about each other’s current approaches to epidemiological applications of information theory.

This opportunity arises especially where there turns out to be an overlap of interests between the two main groups of contributors. For example, Benish’s review provides detailed insight into the analysis of diagnostic information via pre-test probabilities and the corresponding post-test probabilities (predictive values). This theme is then pursued further by means of the predictive receiver operating characteristic (PROC) curve, a graphical plot of positive predictive value (PPV) against one minus negative predictive value (1−NPV) [3,4,5]. Although this format recalls the familiar receiver operating characteristic (ROC) curve, the dependence of the PROC curve on pre-test probability has made it more difficult to characterize and deploy. The articles presented here contribute to an improved understanding of the way that ROC and PROC curves can jointly contribute to the analysis of diagnostic information. An alternative approach to the diagrammatic analysis of diagnostic information via pre-test and post-test probabilities is presented in [6] and then taken up for practical application in [7].

Four articles in the Special Issue apply information-theoretic methods to analyze various aspects of epidemic dynamics [8,9,10,11]. Here, the balance is tipped towards contributions from clinical epidemiology, but information-theoretic applications of time series analysis are presented from both clinical and phytopathological perspectives. Epidemic analyses of observational studies of course depend on the availability of appropriate sample data. In this context, Dalton et al. [12] address the limitations of statistics used to assess balance in observational samples and present an application of the Jensen–Shannon divergence to quantify lack of balance.

Together, the authors whose contributions are presented in this Special Issue have provided a range of novel information-theoretic applications of interest to epidemiologists and diagnosticians in both medicine and plant pathology. While these articles represent the current state of the art, this Special Issue represents only a beginning in terms of what is possible. 
